# Investigation of the feasibility of a simple method for verifying the motion of a binary multileaf collimator synchronized with the rotation of the gantry for helical tomotherapy

**DOI:** 10.1120/jacmp.v13i1.3700

**Published:** 2012-01-05

**Authors:** Masatoshi Hashimoto, Masahiro Uematsu, Makiko Ito, Yukihiro Hama, Takayuki Inomata, Masahiro Fujii, Teiji Nishio, Naoki Nakamura, Keiichi Nakagawa

**Affiliations:** ^1^ Division of Radiology and Biomedical Engineering, Graduate School of Medicine The University of Tokyo Bunkyo‐ku Tokyo Japan; ^2^ Department of Radiology Tokyo Edogawa Cancer Center, Edogawa Hospital Edogawa‐ku Tokyo Japan; ^3^ Department of Radiology International University of Health and Welfare Atami Hospital Atami‐shi Shizuoka Japan; ^4^ Department of Radiology Shinshu University Hospital Matsumoto‐shi Nagano Japan; ^5^ Particle Therapy Division Research Center for Innovation Oncology, National Cancer Center Hospital East Kashiwa‐shi Chiba Japan; ^6^ Department of Radiation Oncology St. Luke's International Hospital Chuo‐ku Tokyo Japan

**Keywords:** helical tomotherapy, verification, multileaf collimator, plastic scintillator

## Abstract

In this paper, we suggest a new method for verifying the motion of a binary multileaf collimator (MLC) in helical tomotherapy. For this we used a combination of a cylindrical scintillator and a general‐purpose camcorder. The camcorder records the light from the scintillator following photon irradiation, which we use to track the motion of the binary MLC. The purpose of this study is to demonstrate the feasibility of this method as a binary MLC quality assurance (QA) tool. First, the verification was performed using a simple binary MLC pattern with a constant leaf open time; secondly, verification using the binary MLC pattern used in a clinical setting was also performed. Sinograms of simple binary MLC patterns, in which leaves that were open were detected as “open” from the measured light, define the sensitivity which, in this case, was 1.000. On the other hand, the specificity, which gives the fraction of closed leaves detected as “closed”, was 0.919. The leaf open error identified by our method was −1.3±7.5%. The 68.6% of observed leaves were performed within ± 3% relative error. The leaf open error was expressed by the relative errors calculated on the sinogram. In the clinical binary MLC pattern, the sensitivity and specificity were 0.994 and 0.997, respectively. The measurement could be performed with −3.4±8.0% leaf open error. The 77.5% of observed leaves were performed within ± 3% relative error. With this method, we can easily verify the motion of the binary MLC, and the measurement unit developed was found to be an effective QA tool.

PACS numbers: 87.56.Fc, 87.56.nk

## I. INTRODUCTION

The demand for the use of high‐technology in radiation therapy is rapidly increasing. In order to concentrate the radiation dose in the tumor, the use of intensity‐modulated radiation therapy (IMRT)^(^ ^1^ ^–^ ^4^ ^)^ has become more widespread. With the technological advances made in IMRT, it has become possible to deliver more complex radiation fields to the target; however, simultaneous verification of the appropriateness of the radiation field also needs to be done. Verification using ion chambers and film is common, and these are well known quality assurance (QA) tools,^(^ ^5^ ^)^ but are basically used for measuring the cumulative dose or radiation field.

New IMRT methods using dynamic multileaf collimators (MLC)^(^ ^6^ ^–^ ^9^ ^)^ and helical tomotherapy (TomoTherapy Inc., Hi·Art, Madison, WI)^(^ ^10^ ^–^ ^12^ ^)^ have been developed and these are now used worldwide. In these methods, the MLC is moved during irradiation and, therefore, its motion must be very precisely controlled. To accurately measure the MLC motion, dynamic observations of it need to be made; however, commercial products^(^ ^13^ ^–^ ^16^ ^)^ for this are generally too expensive. Thus, it has been very difficult to perform such measurements in most treatment facilities or hospitals. Helical tomotherapy, composed of a small 6 MV linear accelerator rotating on a slip ring together with a binary MLC, enables us to deliver a complex dose distribution. The motion pattern of the binary MLC needs to be synchronized with the rotation of the gantry. However, a tool for measuring such motion has not been readily available.

To address this shortcoming, we used a cylindrical plastic scintillator for measuring dose. The classical way of taking measurements with a scintillator is to use a photomultiplier tube (PMT) attached to the scintillator in a light‐proof box.^(^ ^17^ ^)^ Furthermore, Beddar et al.^(^ ^18^ ^,^ ^19^ ^)^ reported that it is possible to measure high‐energy photons and electrons with the detector made of the PMT attached with optical fibers. More recently, built‐in charge‐coupled device (CCD) cameras and optical fibers have been used.^(^ ^20^ ^,^ ^21^ ^)^ Special skills and knowledge are required to build these units. We have developed a similar method that is much simpler to implement. In our method, we use a general‐purpose camcorder instead of a CCD unit. The camcorder is used to record the light image from the scintillator. This method has previously been used for measuring the range of proton and carbon particles and also for QA in brachytherapy and diagnostic computed tomography.^(^ ^22^ ^–^ ^30^ ^)^ We investigated combining these simple devices to measure a complex IMRT field, and also examined the feasibility of using this as a tomotherapy QA technique.

## II. MATERIALS AND METHODS

### A. System setup for measurement

The scintillator used was a cylindrical plastic scintillator (20 cm in diameter by 10 cm in length, Rexon Components, Inc., RP‐400, Beachwood, OH), composed of H and C only, with a density of $1.302\;g/{\text{cm}}^{3}$. The benefits of plastic scintillators are that they have quicker rise times and shorter decay times than inorganic scintillators. The refractive index was 1.58, the rise time, 0.9 nsec, and the decay time, 2.4 nsec.

**Figure 1:**
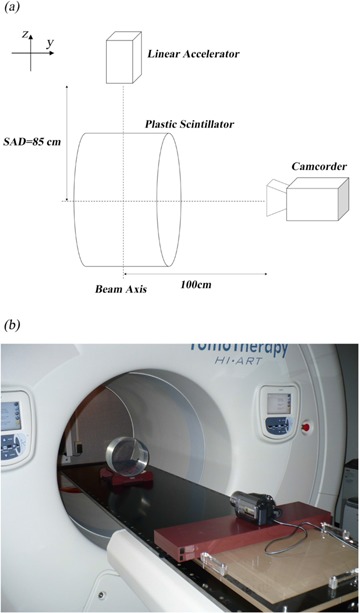
Schematic (a) and photograph (b) of the measurement setup. The center of the plastic scintillator is irradiated with X‐rays. The coordinate system follows the Left‐hand coordinate system. SAD in this figure stands for the source–axis distance.

Figure 1 shows the measurement setup. The plastic scintillator was placed in a helical tomotherapy gantry so that the center of the scintillator was aligned to the isocenter of the gantry. Megavoltage computed tomography (MVCT) was used to precisely position the scintillator. A camcorder (Sony corp., HDR‐HC7, Tokyo) was set at a distance of 100 cm from the isocenter. The camcorder was connected to a personal computer through an IEEE cable, and the scintillation light was recorded as 8‐bit gray scale, 640 × 480 resolution images at 29.97 frames per second (fps). It was previously found that recording an 8‐bit gray scale image would be sufficient for this QA feasibility study.^(^ ^23^ ^–^ ^25^ ^)^ Other settings (e.g., zoom, focus, and sensitivity) were left unchanged. During recording, the room was made as dark as possible. At 29.97 fps, an image can be taken every 33 msec. Under these conditions, our system was able to detect the light and to verify the dynamic motion of the binary MLC and the motion of the rotating gantry. The detected light was converted into image datasets for each and every frame and used for analysis.

**Figure 2:**
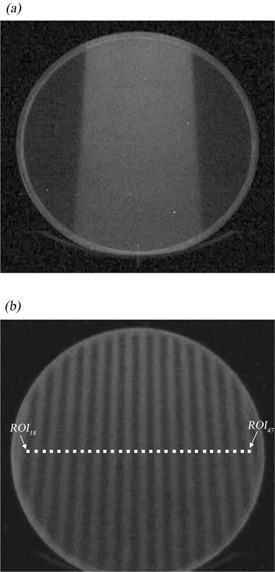
Scintillation light emitted from a $2.5\;\times \;10\;{\text{cm}}^{2}$ field (a), and scintillation light emitted in the case in which the even number leaf positions are open (b). The white boxes are ROIs used to measure the detected light. The ROIs are set at the center of the leaves.

Due to the geometrical limitations of the 20 cm diameter scintillator, it was possible to monitor the radiation field only from leaf number 18 to leaf number 47. Figure 2(a) shows the image from the scintillator of a 6 MV X‐ray collimated radiation field of 2.5 cm × 10 cm.

Figure 2(b) shows the image with the even number collimators opened. For measurement of the light, regions of interest (ROI) of 5 × 5 pixels ($1\;\text{pixel}\;=\;0.468\;\times \;0.468\;{\text{mm}}^{2}$) have been placed on each area corresponding to each leaf number in the image. The average pixel value in the jth ROI of the ith frame is defined as $q_{\text{raw}\;\text{ji}}$, and the quantity of the light detected in each frame was calculated by subtracting the background value, BG, from $q_{\text{raw}\;\text{ji}}$ in each ROI:

(1) $$q_{\text{ji}}(\text{pixel}\;\text{value})=q_{\text{raw}\;\text{ji}}-\text{BG}$$

Accumulating $q_{\text{ji}}$ over the sampling frames, we get

(2) $$Q_{j}\;(\text{pixel}\;\text{value})=\sum_{i}q_{\text{ji}}$$

We use Q for the amount of light measured over all frames and ROI positions. BG is obtained from the average $q_{\text{raw}\;\text{ji}}$ measured in nonexposure (e.g., $q_{\text{raw}\;\text{ji}}$ averaged over 9000 frames was 0.36). This was used as the value of BG in this study.

Under these measurement settings, photons scattered from the collimator rarely, but sometimes, interact with the camcorder and give rise to noise. Such noise is called transient noise, and it is possible to reduce this noise by surrounding the camcorder with radiation shielding material. It is also possible to correct for noise in the images by applying a spatial filtering algorithm (e.g., a median filter). However, it turns out that the filtering correction alters each pixel value.^(^ ^31^ ^)^ In our preclinical tests on the scintillation light detection system, we exposed the scintillator to a 2.5 cm × 10 cm field of 6 MV X‐rays and recorded the light for 300 sec (equivalent to 9000 frames). We observed noise 18 times in the 9000 images, which indicates the probability of transient noise to be 0.2% per frame. This is lower than the noise in similar systems using CCDs. Therefore, for these measurements, neither image filtering nor radiation shielding for noise correction was done.

### B. Characteristics of light detection

In order to verify implementation of the unit as a QA tool, we conducted some basic measurements which are performed in the QA procedure for helical tomotherapy. For the first step, these were done under static field conditions with the gantry angle at 0° and exposure to 6 MV X‐rays at a dose rate of 839 cGy/min.

#### B.1 Relationship between the scintillation light and the collimator open time

It is important to understand the characteristics of the light detected from this system in order to be able to use it to predict the time for which the collimator is open (defined as “leaf open time”). First, we investigated the relationship between the light and the leaf open time from the measurements. The field size was 2.5 cm × 10 cm (opening leaf numbers 25–40). We changed the leaf open time from 29.41 msec to 294.12 msec and made measurements in each case. For these measurements, the value of Q at the isocenter, $Q_{\text{center}}$, is defined as a reference point. Since the isocenter for tomotherapy is located between leaf numbers 32 and 33, this was calculated from the following:

(3) $$Q_{\text{center}}\;(\text{pixel}\;\text{value})=(Q_{32}+Q_{33})/2$$

#### B.2 Lateral profile in the scintillator

The lateral profile in the plastic scintillator is different from the one given by a conventional measurement (e.g., film or 2D profile detector and 3D water measurement), due to the cylindrical shape of the plastic scintillator. Thus, a reference profile for the cylindrical shape is required. This was done by opening a single leaf and exposing the detector to 6 MV X‐rays for 294.12 msec. This process was repeated for all the leaves from 18 to 47, during which time the camcorder was recording the light continuously. Subsequently, we obtained measurements for each $Q_{j}$ (j=18 to 47).

#### B.3 Field size dependency of the detected light

The profile measurement was also performed for several field sizes, where the effective field size ranged from 0.625 cm to 17.5 cm. The exposure time was 294.12 msec. We observed each $Q_{j}$ value, and also looked at the output factor on the central axis.

#### B.4 Exposure time and the detected light

The field size was 10 cm with the 25th–40th leaves opened. The exposure time was 300 sec. The $q_{\text{center}\;i}$ value for each frame was observed frame by frame. We also performed measurements with an ion chamber (Standard Imaging, Inc., A12, Middleton, WI) and a Tomotherapy Electrometer Measurement System (TomoTherapy Inc., Madison, WI). The chamber was placed at the center at a depth of 10 cm in a solid phantom in the same field. The sample time for the ion chamber was 250 msec.

### C. Gantry rotation speed measurement (rotational stability)

The benefit of measurements using the cylindrically shaped detector is the capability of performing dynamic measurements of the rotating gantry in a helical tomotherapy unit. In this measurement, the scintillation light signals are recorded during the gantry rotation, and by observing these we verified if the gantry rotation speed varies at each gantry angle. An X‐ray field along the central axis was established by opening either leaf 32 or 33, and the gantry speed was set to rotate at from 15 to 60 sec per revolution. The narrow beam profiles were recorded frame by frame, from which we observed the position of the beam profile whose side passed through the isocenter.

### D. Binary MLc QA using the cylindrical scintillator

Helical tomotherapy requires a sinogram file to control the motion of the binary MLC. The file consists of each leaf number expressed in the horizontal axis and its corresponding projection number in the vertical axis. In the sinogram, the leaf open times of each leaf number for each projection are given by a number between 0.0 and 1.0. Here 0.0 means that the leaf is closed. For helical tomotherapy, 51 projections can be delivered in one gantry rotation. Suppose one complete rotation of the gantry takes 15 seconds, then one projection needs 294.12 msec (equivalent to 15 secs/51). Therefore, the leaf open time is expressed by the fraction of 294.12 msec. For example, 0.5 expressed in the sinogram means that the leaf open time is 147.06 ms, which is half of 294.12 msec. In a clinical situation, the minimum leaf open time is about 20 msec.

The feasibility of using the scintillator unit as a QA tool was investigated. We attempted to perform the binary MLC QA using the measured $q_{\text{ji}}$. The sinogram was reconstructed from the measured $q_{\text{ji}}$ and was compared with the original sinogram dataset. $q_{\text{ji}}$ is the detected light per frame. The sinogram represents the leaf open time for each projection. In order to reconstruct the sinogram, $q_{\text{ji}}$ needs to be summed for each projection. The summed $q_{\text{ji}}$ is converted into the leaf open time. For the conversion, we used the relationship between the detected light and the leaf open time, which is made based upon the measurement at the center of the plastic scintillator (see Section B.1 above). In this relationship, $q_{j}$ needs to be normalized because if each $q_{j}$ (acquired under the condition that each leaf is open for a certain time) is different from each other, it leads to error. There is another cause of error in that the detected light changes due to exposure time. This also needs to be handled. In this study, two correction factors are applied for each leaf position: one is for correcting the difference in the detected light, $k_{1j}$, and the other is for correcting time variances, $k_{2i}$. The corrected value of the detected light is defined as $q_{\text{cji}}$ as follows:

(4) $$q_{\text{cji}}(\text{pixel}\;\text{value})=q_{\text{ji}}\cdot k_{1j}\cdot k_{2i}$$

Here, $k_{1j}$ and $k_{2i}$ are the lateral profile of the jth ROI and the exposure time correction for the ith frame mentioned in previous sections. In the scintillator and camcorder unit, there may be some issues with respect to the detection of light scattered in the medium and Cherenkov radiation. These phenomena can cause spurious signals in nonexposed ROIs and, in order to deal with these, we set a threshold (Th) to the ROI signal for the scintillation light measurement. Ideally, the threshold values for each ROI bin are derived from the field size effect (output factor in each ROI area) discussed in Section B.3 above. For the purpose of a much simpler verification method, we attempted to determine a general threshold value from the descriptive field size effect measured at center of the field. The threshold value was determined by a receiver operating characteristic (ROC) curve. The $q_{\text{cji}}$ is finally normalized in order to agree with the real exposure time in the original sinogram using the relationship between the exposure time and q measured in section Section B.1 above.

The measurement was performed with the gantry rotating. The gantry was rotated at a constant velocity of 15 sec per revolution. Dose distributions corresponding to the binary leaf patterns show up on the scintillator surface, and the camcorder records these at each gantry angle.

Since the ROIs put on the image in Fig. 2 are fixed at each position and these do not rotate with the gantry rotation, it is not a real‐time measurement. Following the light acquisition for all gantry angles, each of the images acquired are rotated back to 0°, by which we measured the $q_{\text{ji}}$ at each gantry angle. The actual appropriate gantry angles to rotate the images back were given from the result of Section C above. In this way, it is possible to conduct a real‐time binary MLC QA with the rotating gantry during treatment. This is the benefit of this measurement. In the procedure, two sets of sinograms were used. One was a very simple sinogram in which all of the binary MLCs were open for 294.12 msec and this was repeated for 133 projections (simple binary MLC pattern). Another set was the clinical case of a prostate treatment MLC pattern, with 643 projection data and a modulation factor of 1.649 (clinical binary MLC pattern). The modulation factor is defined by maximum leaf open time divided by the average leaf open time. When the modulation factor increases, the leaf open time becomes shorter on the sinogram. In the measurements, the treatment couch was not moved and the number of projections was fixed at 51. The sinogram was analyzed based on the Th value. When $q_{\text{cji}}$ was greater than Th, the jth leaf was identified as being open. When $q_{\text{cji}}$ was less than Th, the jth leaf was considered to be closed. The suitability of the Th value was also evaluated by considering the sensitivity and specificity for some sets of Th values. The leaf open time obtained from this method was compared with the one for the original sinogram data.

## III. RESULTS

### A. Characteristics of light detection

#### A.1 Relationship between scintillation light and collimator open time

**Figure 3:**
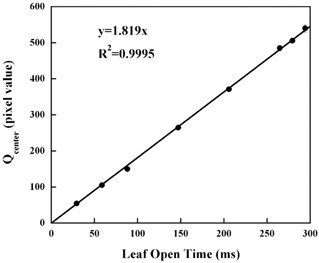
Relationship between the leaf open time and detected light. $Q_{\text{center}}$, on the vertical axis, is calculated from Eq. (3).

The relationship between the detected light Q at the central beam axis, between the 32nd and 33rd leaf positions, is shown in Fig. 3. Good linearity can be seen for leaf open times from 29.41 msec to 294.12 msec. This has already been reported,^(^ ^25^ ^,^ ^29^ ^)^ and we were able to obtain a similar result. The relationship shows that by changing the leaf open time, we can control the exposure to the scintillator and, conversely, that it is possible to predict the leaf open time from the detected light Q.

#### A.2 Lateral profile in the scintillator

**Figure 4:**
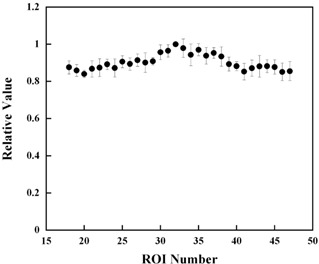
Measured light on each ROI in the case in which each leaf was open individually. These are normalized by the light on the 32nd ROI.

The result of the lateral profile using the scintillator unit is shown in Fig. 4. There is a slight slope in the profile from the center of the beam line towards the off‐axis direction. The main reasons for this are that there is no flattening filter in the linear accelerator used for helical tomotherapy, and the depth doses at each ROI position are different in the cylindrical scintillator. Each value, q, is normalized to the 32nd detected light value, $q_{32}$, which is at the center of the field. The inverse value of the relative value in the profile is used for $k_{1j}$.

#### A.3 Field size dependency of the detected light

**Figure 5:**
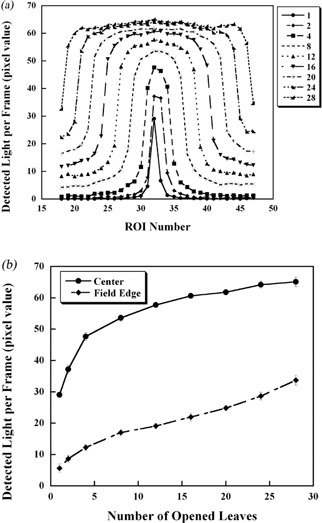
Field dependency of the measured light: the light measured at each ROI and its corresponding leaf position (a); the number of open leaves and the output factor at the central axis and in the field edge region (b). The vertical axis is the detected light per frame.

The relationship between the field size and the beam profile is shown in Fig. 5(a). The $Q_{j}$ in each ROI increases as the field size is widened. This is caused by scattered photons, light scattering^(^ ^25^ ^,^ ^29^ ^)^ and Cherenkov radiation^(^ ^26^ ^,^ ^32^ ^,^ ^33^ ^)^ in the scintillator. Figure 5(b) shows the output factors in terms of $Q_{j}$ at the center and at the field edge for each field size in Fig. 5(a). The center is at the 32nd leaf position when opening one leaf, and between leaves 32 and 33 when opening two or more leaves. The Q at the field edge is defined, when the leaves from j to j+n are open, as the average value of $Q_{j-1}$ and $Q_{j+n+1}$. For example, when just the 32nd leaf is open, the field edge is the average of $Q_{31}$ and $Q_{33}$, and when the 32nd–33rd leaves are open, the field edge is the average of $Q_{31}$ and $Q_{34}$. The detected light per frame at center increases from 29.1 up to 65.1 pixel value. In the field edge, the detected light per frame increased from 5.6 to 33.7 pixel value. The light detected at the center when just one leaf was open is lower than the light detected at the field edge when 24 leaves were open (Fig. 5(b)). This means that, in the case of the wider field shown in Fig. 5(a), even if a few leaves are not open at the side, the unopened leaves might possibly be recognized as being “open”. This might be the cause of errors in this measurement.

#### A.4 Exposure time and the detected light

**Figure 6:**
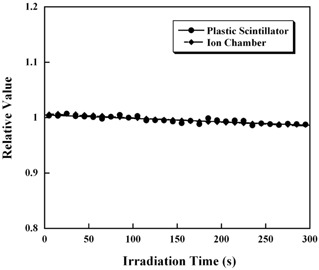
Stability of the data measured with an ion chamber and the plastic scintillator. The measured data from the scintillator are based on $q_{\text{center}\;i}$. The data from the ion chamber are from measurements made at a depth of 10 cm. Each plot is averaged over a time of 10 sec and normalized by the value at 100 sec. The readings of both the plastic scintillator and ion chamber decreased with exposure time.

The relationship between irradiation time and the scintillation light detected (relative value) on the camcorder is shown in Fig. 6. The detected light is slightly greater than 1 for short irradiation times, and then gradually decreases. Since this trend is also seen in the ion chamber measurements, it is thought that the slight variation was due to a variation in the beam output and not to the scintillator. However, since the variation in the beam output affects the results, a correction factor $k_{2i}$ for the beam output based on this curve was applied.

### B. Gantry rotation speed measurement (rotational stability)

**Figure 7:**
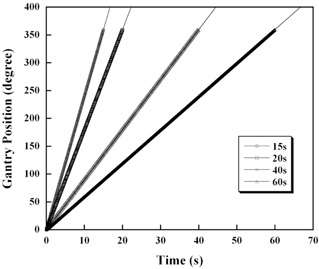
Stability of the gantry rotational speed. The solid axis represents the theoretical value.

Figure 7 shows the gantry position (degree) and the time from the gantry rotation start position. The solid lines in the figure express the theoretical values on the basis of the assumption that the gantry rotational speed is constant. There is good agreement between the measured and theoretical values for rotational speeds of 15 to 60 sec/revolution, and these are constant for all rotational speeds. Thus, regarding the appropriate gantry angles for rotating back the images, it turned out that the nominal gantry rotation angles are useful.

### C. Binary MLc QA using the cylindrical scintillator

**Figure 8:**
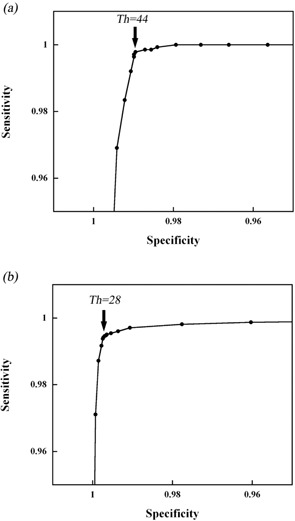
ROC curves for a simple binary MLC pattern model (a) and a clinical binary MLC pattern model (b). The Youden index is at its maximum value at Th=44 pixel value in the simple model and at Th=28 pixel value in the clinical model.

Figure 8 shows the ROC curves for the simple model and the clinical model. Setting the threshold, Th, too high, would give a false value. Setting the threshold too low results in spurious signals being detected on each ROI. This is also affected by the leaf open time. It is important, therefore, for us to consider the optimum Th value to make an adequate balance between sensitivity and specificity. Therefore, we employed the Youden index,^(^ ^34^ ^)^ which is calculated from “sensitivity+specificity−1” and ranges from 0 to 1. We believe that the maximum Youden index represents the optimum Th in this case. From the results of the simple model, the maximum value is 0.987 when Th is at the 44 pixel value, with the sensitivity being 0.998 and the specificity 0.989. According to Fig. 5, Th=44 pixel value is for the case for which between 2 and 4 leaves are open. However, in the case of one leaf only open, the detected light per frame is 29.1 pixel value (Fig. 5). Thus, if the value of Th=44 pixel value were to be used, the case of one leaf open might be identified as not open.

In the clinical case, the maximum Youden index is 0.992 at Th=28 pixel value. As shown in Fig. 5, Th=28 pixel value is less than the measured value with one leaf open (29.1 pixel value). In the clinical model, the beam intensity generated from each binary MLC is modulated, even though leaves neighboring each other are open; it is not uncommon for each leaf open time to be different. Looking at the leaf open times frame by frame (every 33 msec), we can see when only one leaf is open. Therefore, we consider that Th=28 pixel value, with which we are able to detect even one leaf open, is the most appropriate value. From the observations, the appropriate Th itself actually varies depending on the variation in the leaf pattern. Nevertheless, we believe that Th=28 pixel value is the appropriate value because with it, the one leaf open status can be detected and its flexibility makes it applicable for any leaf pattern.

**Figure 9:**
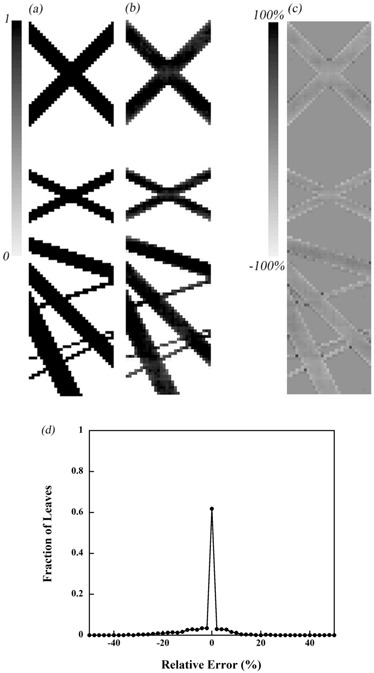
Sinogram for planned simple binary MLC pattern model (a), measured sinogram (b), difference between these sinograms (c), and the resulting histogram (normalized) (d).

Figure 9(a) shows the leaf open patterns expressed as original sinogram. Figure 9(b) is the reconstructed sinogram from measured projection datasets. The sensitivity and specificity are 1.000 and 0.919, respectively. Figure 9(c) shows the relative error. The relative error was calculated from the difference between Fig. 9(a) and Fig. 9(b), and was divided by the maximum value for each projection. Figure 9(d) represents a histogram of the values in Fig. 9(c). The error associated with the leaf open time is calculated from the mean and standard deviation of the relative errors. The calculation result was −1.3±7.5%, and this is defined as the leaf open error. The 68.6% of all observed leaves were performed within ±3% relative error. In some ROIs, the light measured was above the threshold but the leaf at the corresponding position was not open. This was due to field edge light from other leaf positions pushing the value of $Q_{j}$ over the threshold value. This was very obvious in the case of ${\text{ROI}}_{j}$ with the leaf numbers j+1 and j−1 open and j closed.

**Figure 10:**
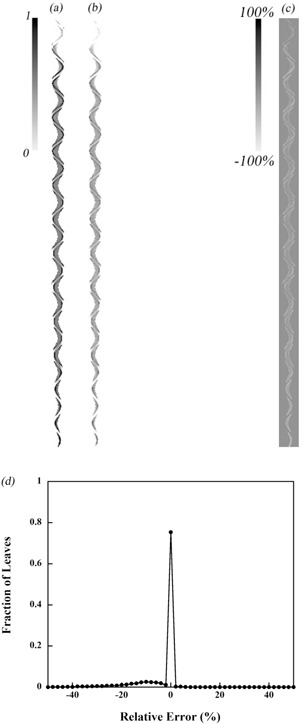
Sinogram on planned clinical binary MLC pattern model (a), measured sinogram (b), difference between these sinograms (c), and the resulting histogram (normalized) (d).

The sinogram clinically used for a prostate cancer patient is shown in Fig. 10(a); Fig. 10(b) is the reconstructed data from the measurement. The sensitivity and specificity are 0.994 and 0.997, respectively. The leaf open error was −3.4±8.0%. The 77.5% of observed leaves were performed within ±3% relative error. With respect to the errors, the status that some leaves were open but not recognized as open occurred at a given leaf position because Q was below the threshold value. Conversely, the case in which leaves were not open but were recognized as open occurred around the same area of the simple sinogram result.

## IV. DISCUSSION

The leaf open errors were −1.3±7.5% for the simple model and −3.4±8.0% for the clinical model. The sensitivity and specificity were > 0.9, which means that the leaf status was correctly recognized in 90% of the cases. Hence the main reason for the detection errors might be caused by use of the formula used to convert detected light to leaf open time, which is based on the results of the 2.5 cm × 10 cm field measurement rather than the value of Th. As already shown in Fig. 5, the detected light changes depending on the field size, even though the leaf open time is constant. In the case of just one leaf being open, the detected light was 0.48 compared to the 10 cm field size. This change becomes more obvious for smaller field sizes. In the clinical model, the field size per frame is generally smaller than the simple field due to the intensity modulated field. This is why the error for the clinical model turned out to be −3.4±8.0%, which is much larger than that for the simple model. Regarding the measured light correction, the light measurements obtained from sets of field sizes were normalized based on the light measured for the 10 cm field. However, for the leaf pattern for the simple model, scintillation light emitted from a corresponding leaf location was scattered in the medium and affected the adjacent leaf positions. The error associated with scattering varies depending on the field size and the scatter. Thus, ideally it would be preferable to calculate a variety of correction factors for any leaf pattern; this, however, is much too complicated.

We used the Youden index as a criterion in order to optimize the value of Th. The Youden index is computed from a comparison between the original sinogram and the sinogram delivered from the measurement. The Th value was originally supposed to be derived from a comparison between the original leaf pattern and the one from the measurement, by which the errors are also estimated. Nevertheless, since reliable measured data could not be obtained, we chose to use the planned sinogram as a reference value.

The errors in this study were caused by not using correction factors for Th depending on field size. If scattered light did not enter neighboring ROIs, no corrections associated with field size would be necessary. In such a situation, we could achieve accurate measurements and the errors would perhaps be independent of the Th value. In order to reduce the scattering, it might be a better solution to collimate the scattered light. Ikegami et al.^(^ ^35^ ^)^ reported that it is possible to collimate the scintillation light and measure 3D dose distributions using scintillation fibers. If we were able to use scintillation fibers, it could be possible to perform our measurement with a higher accuracy.

Nevertheless, we have demonstrated that a simple measuring device using a combination of a camcorder and a cylindrical scintillator can work as a binary collimator QA device without light correction. With this device, measurements can be performed even in a clinical case with a sensitivity and specificity of more than 0.99 and a leaf open error of −3.4±8.0%. Since the sensitivity and specificity are more than 0.99, and this measurement can identify the leaf positions where errors are most likely occur, we believe that this measurement can be used to detect leaf motion where the leaf is open but recognized as not so, or the opposite case.

In this study, we have used 20 cm diameter scintillator. It detected the radiation field only from leaf number 18 to leaf number 47. The binary MLC is composed of 64 leaves, which effectively makes a 40 cm field (in width) at isocenter. It would be preferable to use at least 40 cm diameter scintillator for all measurements. However, if such a detector were to be used for QA, it would be necessary to evaluate if our method would be applicable with a large scintillator size.

Kapatoes et al.^(^ ^36^ ^)^ has reported a similar study where the sinogram is reconstructed using MIMiC MLC (Nomos Corporation, Pittsburgh, PA) and a CT detector, where the possibility of beam delivery similar to helical tomotherapy was investigated. Indeed it could be possible to do a similar trial with a helical tomotherapy unit. However, since the accelerator and CT detector are combined with an actuator and these are not independent, it is impossible to conduct the gantry angle check with this unit. Besides, it is difficult for users to make a use of the signal obtained from a CT detector, which leads to a measurement lacking in versatility. The benefits of our method are that our unit can dynamically measure the motion of the binary leaves at any gantry angle (as well as the gantry position during rotation), and it provides simplicity of measurement. In this study, we accomplished observations of a binary MLC and the gantry angle easily with a simple unit consisting of a cylindrical scintillator and a general‐purpose camcorder. We believe that this presents us with a very feasible QA tool.

## V. CONCLUSIONS

We have developed a simple QA tool that can easily check binary MLC motion. This is composed of a cylindrical scintillator and a widely‐used camcorder. The camcorder can monitor the binary MLC motion via scintillation light. Using the QA tool, we verified a simple binary MLC pattern and a more complicated MLC pattern used in clinic. In the sinogram of the simple binary MLC pattern, the leaves that were supposed to be open were detected with “open” status with respect to the detected light, and the sensitivity was 1.000. On the other hand, the leaves that were not supposed to be open were usually detected as such giving rise to a specificity of 0.919. The measurement was achievable with −1.3±7.5% leaf open error. The 68.6% of observed leaves were performed within ±3% relative error. In the clinical binary MLC pattern, the sensitivity and specificity were 0.994 and 0.997, respectively. The measurement could be performed with −3.4±8.0% leaf open error. The 77.5% of observed leaves were performed within ±3% relative error. These errors accounted for the values that are dependent on the planned leaf pattern. In order to remove such dependency, one needs to correct for the contribution of light scatter, which requires further study. The results of this study demonstrated that it is possible to dynamically detect the motion of a binary MLC, which is a difficult task with conventional film or ion chamber measurement. Our method has been investigated without couch motion, and we conclude that we are able to perform accurate verification with this constraint. Although this method is not a perfect alternative for QA, it is more easily performed than that which uses a combination of ion chamber and film measurements.
